# Validity of the Czech Translation of the Adult Attention-Deficit/Hyperactivity Disorder (ADHD) Self-Report Scale (ASRS)

**DOI:** 10.3389/fpsyg.2022.799344

**Published:** 2022-05-05

**Authors:** Martina Vňuková, Radek Ptáček, Filip Děchtěrenko, Jiří Raboch, Martin Anders, Michal Goetz

**Affiliations:** ^1^Department of Psychiatry, First Faculty of Medicine, Charles University and General University Hospital in Prague, Prague, Czechia; ^2^Department of Mathematics, College of Polytechnics, Jihlava, Czechia; ^3^Department of Psychiatry, National Institute of Mental Health, Klecany, Czechia; ^4^Children’s Psychiatric Hospital Opařany, Opařany, Czechia; ^5^Department of Psychiatry, Third Faculty of Medicine, Charles University, Prague, Czechia

**Keywords:** ASRS, adult attention-deficit/hyperactivity disorder, screener, WURS, validity

## Abstract

**Objective:**

The study aim was to assess the psychometric properties of the ASRS in the Czech Republic. Although this screening tool is now frequently used, its validity has not been assessed among the general Czech population.

**Methods:**

The ASRS and WURS were administered online to the general Czech population (*N* = 1,518). We performed confirmatory and exploratory factor analyses.

**Results:**

For the ASRS, confirmatory factor analysis showed good fit for the screening part (SRMR = 0.03, RMSEA = 0.06, CFI = 0.98). For the symptom list, the fit was good according to the SRMR, acceptable according to the RMSEA, and slightly below acceptable according to the CFI. For the WURS, the results showed SRMR = 0.06, RMSEA = 0.07, and CFI = 0.92.

**Conclusion:**

The Czech translation of the ASRS is appropriate and has acceptable psychometric properties. However, we strongly recommend only using this tool together with clinical judgment.

## Introduction

Attention-deficit/hyperactivity disorder (ADHD) is a neurodevelopmental disorder that is defined as a group of predominantly genetically based deficits characterized by three main domains of symptomatology: inattention, hyperactivity, and impulsivity (American Psychiatric Association, 2013). ADHD is most frequently diagnosed in childhood, with a prevalence of approximately 5%–10% (Kessler et al., 2010; Yallop et al., 2015). However, the DSM 5 recognized ADHD as a lifelong disorder, and it is now known that approximately half of the cases persist into adulthood (Montejano et al., 2011; Ramos-Quiroga et al., 2013). Our recent study of ADHD prevalence in adulthood in a representative sample of the Czech population identified a prevalence of approximately 3.5%, with the prevalence being higher among males than females (Vňuková et al., 2021). ADHD disrupts the daily functioning of affected individuals, and in adulthood, an association has been found with lower school performance or higher rates of unemployment as well as substance abuse and eating disorders (Prihodova et al., 2010; Michielsen et al., 2012; Gupta, 2016; Weissenberger et al., 2018).

Knowledge of ADHD as a disorder that can be diagnosed in adulthood calls for more attention and research in the field of psychodiagnostics (Montejano et al., 2011; Efron, 2017). While ADHD as a childhood disorder is well established and recognized among clinical professionals and while its definition and diagnostic criteria have been developed and adjusted since 1980 (DSM-III), the notion of ADHD as a lifelong disorder is still a fairly new concept (DSM 5 was published in 2013; American Psychiatric Association, 2013). Furthermore, the international classification of diseases 10 (ICD 10) only works with the concept of hyperkinetic disorder, which, unlike ADHD, has stricter symptomatic criteria and is still only a childhood disorder; that is, the symptoms need to occur before the age of six (World Health Organization, 2019).

ADHD symptomatology can be measured and assessed in multiple ways, although the diagnosis can be established only by qualified psychiatrists according to DMS five criteria. The most frequent methods still tend to be structured clinical interviews led by a trained clinician. However, multiple other tools have been developed over the years, such as computer assessment tools and questionnaires for parents, guardians, or teachers, as well as individual retrospective self-assessment and current self-assessment (Knopf et al., 2012; Knopf, 2018; Weibel et al., 2020). There may be disparities among these assessment tools, as they rely on honesty, objective assessment, and often memory recall. It can often be difficult for an individual (or parent/guardian) to correctly assess and recognize the possible presence of ADHD symptoms. Furthermore, the way in which symptoms manifest at a later age may change from their manifestation in childhood. As adults, individuals often learn to cope with their symptoms, and the invented coping systems and strategies that are in place may mask the true manifestation of the underlying symptoms.

One of the self-assessment tools that is most frequently used is the Adult ADHD Self-Report Scale (ASRS v1.1). This tool was originally developed by Kessler and his colleagues from a WHO working group (Kessler et al., 2005). This scale has been found to be a valid screening tool not only among adults but also among adolescents (Green et al., 2019). It is a self-report scale that is scored based on symptom frequency. This screening tool focuses on the current symptoms, and their identification is crucial for ADHD diagnosis in adulthood. A short 6-item version was created from the full 18-item ASRS (Kessler et al., 2005). To capture the multidimensional nature of ADHD, the ASRS-v1.1 has two subscales: inattention and hyperactivity/impulsivity. The ASRS has been found to have good validity and reliability, and it has been tested among different cultures as well as in different languages (Wyrwich et al., 2016; Kiatrungrit et al., 2017; Takeda et al., 2017; Somma et al., 2019). Furthermore, it has been shown that the short screening version can be used in the general population (Carlucci et al., 2017; Silverstein et al., 2018; Green et al., 2019). Overall, it also outperformed the longer 18-item version of the questionnaire (Kessler et al., 2007a). The ASRS has therefore been recognized as a good screening tool for ADHD, and its use has been recommended (Adler et al., 2006; Green et al., 2019). ASRS has also been identified as a tool with very few missed cases among populations with substance use disorder (Van de Glind et al., 2013). In the United States, there have even been attempts to establish norms for the ASRS results (Adler et al., 2019).

Since 1980, when ADHD was first conceptualized, we have seen a shift and a development of this concept from a purely childhood disorder to a lifelong disorder that can affect almost all areas of life and daily functioning and can even have a negative impact on the social circle of the affected individual (Ptacek et al., 2014b; Weissenberger et al., 2018). In the Czech Republic, a lack of acceptance of ADHD as a lifelong disorder has generated many challenges. The transition toward lifelong disorder means that there might be adult individuals who never received ADHD diagnosis and adequate help due to the lack of proper tools for diagnosis and understanding how the symptoms change and transform as the individual progresses in life. Not only the new diagnostic manual, such as ICD 11, but also adequate tools for diagnosis might guarantee a deeper understanding of this symptomatologic transition, which is still lacking.

This lack of understanding demonstrates the need for our study, as the ASRS has been translated into the Czech language, but the validity and reliability of this screening tool translation have yet to be explored. The aims of the present study were primarily to examine the psychometric properties of the Czech translation of the ASRS using the Czech translation of the Wender Utah Rating Scale (WURS) in the general population (Kessler et al., 2005, 2007b).

## Materials and Methods

The current study is a part of the research project supported by the Grant Agency of the Czech Republic entitled “ADHD and Perception of Time” and was approved by the ethics committee of the General University Hospital, Prague, Czech Republic. The data were collected through the computer-assisted web interviewing (CAWI) method, that is, an online survey, with respondents from the European National Panel. For a detailed description of the methodology and the study sample, see Vňuková et al. (2021). The exclusion criteria were as follows: (1) presence of severe neuropsychiatric disorder (especially intellectual disability, schizophrenia, psychosis, severe mood disorder, dementia, substance abuse or behavioral dependence, or neurodegenerative diseases); (2) severe somatic disorders with a direct effect on cognitive/executive functions (especially cardiovascular, cerebrovascular or endocrine diseases); and (3) use of drugs that potentially affect cognitive function. This information was included in the informed consent that the participants had signed prior to the research. As this was a cross-sectional online study, we did not have access to participants’ medical records or collected information about their current medications. We relied on their self-reported answers that they were not receiving any psychopharmacological treatment at the moment.

A total of 1,518 completely completed questionnaires were collected. The panel of respondents constituted a representative sample of the population of the Czech Republic according to age, sex, education, and place of residence. Participants were approached by the research company STEM/MARK, which ensures representativeness based on quotas that are created according to the census of the Czech population.

Respondents completed a demographic questionnaire focusing on the history of ADHD, risky behavior, and lifestyle. The test battery also included two standardized questionnaires focusing on ADHD symptomatology: the WURS for ADHD retrospective symptomatology in childhood and the ASRS mapping the current symptomatology of ADHD in adulthood.

The ASRS (Kessler et al., 2005) is an 18-item scale based on ADHD criteria according to DSM IV TR and DSM 5. It was created by the working group for ADHD at the WHO. The presence of ADHD symptomatology is evaluated on a 5-point Likert scale (*never, rarely, sometimes, often, very often*) with a total score ranging from 0 to 72; symptoms are assessed retrospectively over the past 6 months. Furthermore, the authors recommend using only the shortened version, that is, the first six questions; with the shortened version, either the sum score of all questions can be computed, or the answers in part A that are scored as often-very often can be identified. Good psychometric characteristics of this scale have been reported (Cronbach’s α = 0.88–0.89; Adler et al., 2006). The WURS (Ward et al., 1993) is a 25-item self-assessment scale aimed at retrospectively assessing the presence of ADHD symptoms in childhood in adults. It is recommended in the current literature as a reliable tool for this purpose (Taylor et al., 2011), with good discriminative ability (McCann et al., 2000). It was translated into the Czech language by Paclt (2002), and it has been recommended for use in combination with clinical interviews, as it is an objective retrospective screening tool for ADHD symptomatology. It has been further used or recommended as a screening tool in adulthood by other research groups (e.g., Paclt, 2007; Cahová et al., 2010; Rubášová et al., 2015). However, to date, there has been no larger-scale study among Czech adults.

### Data Analysis

Data were analyzed in *R* statistical software (R Core Team, 2020). Confirmatory factor analysis (CFA) was performed using the *lavaan* package (Rosseel, 2012), while exploratory factor analysis (EFA), including item analysis, was conducted using the *psych* package (Revelle, 2022). For the CFA, we evaluated the fit using common indices as suggested by Hu and Bentler (1999). According to their research, fit is considered to be good if the standardized root mean square residual (SRMR) is lower than 0.08, the root mean square error of approximation (RMSEA) is lower than 0.06, and the comparative index fit (CFI) is larger than 0.95. The fit is considered to be acceptable if the RMSEA is lower than 0.08 and the CFI is larger than 0.9. In the EFA, we estimated the number of factors using the consensus method from the *parameters* package (Lüdecke and Waggoner, 2019). This method runs multiple commonly used methods for assessing a number of factors (such as Keiser’s criteria, Velicer’s minimum average partial, or very simple structure) and selects the number of factors on which the majority of the methods agree. Following the selection of a number of factors, we randomly divided the data into two subsamples. For the first subsample, containing 70% of the data (*n* = 1,062), we performed EFA with the number of factors estimated by the previous step. Based on the results of the EFA, we performed CFA with the suggested factor structure on the second subsample (containing 30% of the data, *n* = 456). To estimate the reliability, we computed both the traditional Cronbach’s alpha and the omega total. Although the omega total gives better estimates of reliability at the population level, we used Cronbach’s alpha for item analysis, as both estimates yielded similar results for the whole scale and as obtaining Cronbach’s alpha at the item level was computationally easier. In the case of the ASRS questionnaire, we analyzed the screening part separately in addition to all items, as commonly done in other validation studies (Kessler et al., 2005, 2007a). We tested the validity of the ASRS using the WURS. We showed the correlation between the ASRS and WURS for the whole sample and examined the stability of these correlations for different demographic subgroups, namely, by sex, age category, and education category. We did not have data regarding clinical assessment, that is, whether respondents had ADHD; thus, for the ASRS, we computed three measures: screening total (sum of points in the screening part), screening score (0–6 points based on the response patterns), and symptom list total (sum of all 18 items).

## Results

For the ASRS, CFA showed good fit for the screening part: *χ*^2^(8) = 51.3, *p* < 0.001, SRMR = 0.03, RMSEA = 0.06, CFI = 0.98, TLI = 0.96. For the symptom list, the fit was good according to the SRMR, acceptable according to the RMSEA, and slightly below acceptable according to the CFI. As suggested Lai and Green (2016), in conditions when several indices disagree, further analysis of the model structure is needed. We thus proceeded to EFA of the sample. First, the consensus method indicated that either one- or three-factor solutions were assessed as the most valid (5 out of 23 methods). Running EFA on the first subsample followed by CFA on the second subsample yielded below acceptable fit (*χ*^2^(135) = 713.4, *p* < 0.001, SRMR = 0.07, RMSEA = 0.10, CFI = 0.79, TLI = 0.76) based on the CFI and other fit indices. A similar approach for the three-factor solution resulted in an acceptable fit (*χ*^2^(132) = 468.5, *p* < 0.001, SRMR = 0.06, RMSEA = 0.07, CFI = 0.88, TLI = 0.86) according to all fit indices. The final loadings and division into factors are displayed in Table 1.

**Table 1:** ASRS factor loadings.

Variable		f1	f2	f3	Communalities	Uniqueness
Inattentive						
ASRS 1	How often do you have trouble wrapping up the final details of a project, once the challenging parts have been done?	**0.63**	0.24	0.11	0.47	0.53
ASRS 2	How often do you have difficulty getting things in order when you have to do a task that requires organization?	**0.69**	0.09	0.08	0.49	0.51
ASRS 3	How often do you have problems remembering appointments or obligations?	0.50	0.12	0.27	0.34	0.66
ASRS 4	When you have a task that requires a lot of thought, how often do you avoid or delay getting started?	**0.67**	0.12	0.18	0.49	0.51
ASRS 7	How often do you make careless mistakes when you have to work on a boring or difficult project?	**0.58**	0.22	0.33	0.5	0.50
ASRS 8	How often do you have difficulty keeping your attention when you are doing boring or repetitive work?	**0.62**	0.18	0.35	0.53	0.47
ASRS 9	How often do you have difficulty concentrating on what people say to you, even when they are speaking to you directly?	**0.52**	0.19	0.35	0.43	0.57
ASRS 10	How often do you misplace or have difficulty finding things at home or at work?	0.44	0.12	0.4	0.37	0.63
ASRS 11	How often are you distracted by activity or noise around you?	0.45	0.19	0.4	0.4	0.6
**Hyperactive/impulsive**						
ASRS 5	How often do you fidget or squirm with your hands or feet when you have to sit down for a long time?	0.29	0.13	0.42	0.27	0.73
ASRS 6	How often do you feel overly active and compelled to do things, like you were driven by a motor?	0.07	0.25	0.40	0.23	0.77
ASRS 12	How often do you leave your seat in meetings or other situations in which you are expected to remain seated?	0.25	0.25	0.35	0.25	0.75
ASRS 13	How often do you feel restless or fidgety?	0.4	0.24	**0.54**	0.51	0.49
ASRS 14	How often do you have difficulty unwinding and relaxing when you have time to yourself?	0.15	0.22	0.45	0.28	0.72
ASRS 15	How often do you find yourself talking too much when you are in social situations?	0.03	**0.63**	0.24	0.46	0.54
ASRS 16	When you are in a conversation, how often do you find yourself finishing the sentences of the people you are talking to, before they can finish them themselves?	0.14	**0.67**	0.17	0.50	0.50
ASRS 17	How often do you have difficulty waiting your turn in situations when turn taking is required?	0.24	**0.65**	0.24	0.54	0.46
ASRS 18	How often do you interrupt others when they are busy?	0.34	**0.56**	0.17	0.46	0.54

Bold values are to indicate on which factor the given question is loading.

For the WURS, we first ran CFA using a three-factor solution as suggested by Brevik et al. (2020). The fit was below acceptable based on all fit indices; thus, we proceeded to EFA. First, the consensus approach showed that the most likely factor structure was a 4-factor solution. Running the EFA on the first subsample followed by CFA on the second subsample did not show a good fit: *χ*^2^(269) = 1211.3, *p* < 0.001, SRMR = 0.07, RMSEA = 0.09, CFI = 0.84, TLI = 0.82. We thus inspected the modification indices of the model and repeatedly removed the items from the model with the highest modification indices. This led to the removal of seven items (1, 5, 6, 10, 11, 23, 24) until we reached an acceptable fit according to fit indices [*χ*^2^(129) = 442.6, *p* < 0.001, SRMR = 0.06, RMSEA = 0.07, CFI = 0.92, TLI = 0.90]. In Table 2, we show both factor loadings on the first subsample for the four-factor solution, including communalities and uniqueness. Additionally, we also show the factor loadings from the final CFA after we removed items that decreased the fit of the model. Overall, the ASRS showed a better factor structure than the WURS.

**Table 2:** Item analysis (we also show the reliability of the test if the item was dropped and standardized factor loadings from the CFA for the symptom list).

Variable			Mean	SD	Skewness	Alpha if dropped	Correlation with score	Correlation if dropped	Inattention	Hyperactivity impulsivity
Inattentive										
ASRS 1	How often do you have trouble wrapping up the final details of a project, once the challenging parts have been done?		2.21	0.90	0.62	0.88	0.60	0.55	0.64	–
ASRS 2	How often do you have difficulty getting things in order when you have to do a task that requires organization?		2.03	0.89	0.79	0.88	0.56	0.51	0.61	–
ASRS 3	How often do you have problems remembering appointments or obligations?		2.06	0.96	0.79	0.88	0.54	0.51	0.57	–
ASRS 4	When you have a task that requires a lot of thought, how often do you avoid or delay getting started?		2.33	0.95	0.55	0.88	0.59	0.55	0.65	–
ASRS 7	How often do you make careless mistakes when you have to work on a boring or difficult project?		2.43	0.80	0.45	0.88	0.68	0.64	0.72	–
ASRS 8	How often do you have difficulty keeping your attention when you are doing boring or repetitive work?		2.54	0.96	0.41	0.88	0.69	0.64	0.75	–
ASRS 9	How often do you have difficulty concentrating on what people say to you, even when they are speaking to you directly?		2.11	0.91	0.64	0.88	0.66	0.62	0.67	–
ASRS 10	How often do you misplace or have difficulty finding things at home or at work?		2.73	0.98	0.31	0.88	0.55	0.51	0.56	–
ASRS 11	How often are you distracted by activity or noise around you?		2.64	1.00	0.33	0.88	0.59	0.55	0.59	–
**Hyperactive/impulsive**										
ASRS 5	How often do you fidget or squirm with your hands or feet when you have to sit down for a long time?		2.48	1.15	0.44	0.88	0.50	0.47	-	0.48
ASRS 6	How often do you feel overly active and compelled to do things, like you were driven by a motor?		2.34	0.98	0.49	0.89	0.36	0.35	-	0.39
ASRS 12	How often do you leave your seat in meetings or other situations in which you are expected to remain seated?		1.56	0.77	1.39	0.88	0.50	0.47	–	0.54
ASRS 13	How often do you feel restless or fidgety?		2.15	0.89	0.68	0.88	0.68	0.64	-	0.67
ASRS 14	How often do you have difficulty unwinding and relaxing when you have time to yourself?		2.26	1.15	0.61	0.89	0.44	0.42	–	0.47
ASRS 15	How often do you find yourself talking too much when you are in social situations?		2.27	1.04	0.60	0.89	0.45	0.42	–	0.53
ASRS 16	When you are in a conversation, how often do you find yourself finishing the sentences of the people you are talking to, before they can finish them themselves?		2.13	1.00	0.65	0.88	0.51	0.48	–	0.59
ASRS 17	How often do you have difficulty waiting your turn in situations when turn taking is required?		2.12	0.98	0.69	0.88	0.62	0.58	–	0.70
ASRS 18	How often do you interrupt others when they are busy?		1.83	0.80	0.85	0.88	0.60	0.56	–	0.65
**Variable**	**Mean**	**SD**	**Skewness**		**Alpha if dropped**		**Correlation with score**		**Correlation with score if dropped**	
WURS 1	1.90	1.10	1.07		0.93		0.64		0.61	
WURS 2	2.24	1.22	0.67		0.94		0.52		0.50	
WURS 3	2.09	1.16	0.82		0.93		0.70		0.68	
WURS 4	2.03	1.12	0.86		0.93		0.70		0.67	
WURS 5	1.73	1.00	1.26		0.93		0.69		0.66	
WURS 6	1.75	1.05	1.31		0.93		0.64		0.61	
WURS 7	1.91	1.09	1.02		0.93		0.65		0.64	
WURS 8	2.28	1.24	0.60		0.94		0.54		0.52	
WURS 9	1.81	1.08	1.23		0.93		0.67		0.65	
WURS 10	1.89	1.09	1.10		0.94		0.58		0.55	
WURS 11	2.51	1.30	0.44		0.94		0.50		0.48	
WURS 12	1.75	0.99	1.27		0.93		0.76		0.73	
WURS 13	1.79	1.04	1.24		0.93		0.72		0.70	
WURS 14	1.73	0.99	1.30		0.93		0.75		0.72	
WURS 15	1.82	1.06	1.18		0.93		0.70		0.67	
WURS 16	1.75	1.02	1.25		0.93		0.70		0.67	
WURS 17	2.06	1.16	0.86		0.93		0.66		0.64	
WURS 18	1.45	0.85	2.05		0.93		0.68		0.65	
WURS 19	1.56	0.90	1.61		0.93		0.67		0.64	
WURS 20	1.74	1.07	1.44		0.94		0.53		0.51	
WURS 21	1.76	1.00	1.26		0.94		0.58		0.56	
WURS 22	1.61	1.03	1.65		0.94		0.56		0.53	
WURS 23	1.64	0.99	1.48		0.94		0.51		0.49	
WURS 24	1.99	1.20	1.02		0.94		0.43		0.41	
WURS 25	2.17	1.19	0.67		0.94		0.52		0.51	

In Table 3, we show several reliability coefficients. We show the reliability of all items (for both the screening part and the symptom list) followed by the reliability of both scales when computed individually. In the case of screening, hyperactivity consists of only two items, which usually results in lower estimates of reliability. Taken together with the finding that questions five and six showed inconsistent loading to latent factors, using the scales separately should be handled with caution.

**Table 3:** Cronbach’s alpha and omega total for each of the scales.

Scale name	*α*	95% CI	**ω**_T_	95% CI
ASRS total	0.89	[0.88–0.9]	0.89	[0.88–0.9]
ASRS screener	0.73	[0.71–0.74]	0.72	[0.7–0.75]
ASRS screener hyperactive/impulsive	0.49	[0.45–0.52]	–	–
ASRS screener inattentive	0.76	[0.75–0.78]	0.77	[0.75–0.78]
ASRS hyp	0.86	[0.85–0.87]	0.86	[0.85–0.87]
ASRS int	0.79	[0.78–0.81]	0.79	[0.78–0.81]
WURS	0.94	[0.93–0.94]	0.94	[0.93–0.94]

Item analysis for the ASRS is displayed in Table 2 and for the WURS in Table 4. In addition to the mean, SD, and skewness, we also show correlations with the total score, correlations with the total score if the item was dropped, and reliability (estimated by Cronbach’s alpha) if the item was dropped. Additionally, we also show factor loadings from the original CFA.

**Table 4:** Correlation matrix.

	WURS	ASRS_HS	ASRS_total	ASRS_score
WURS	**–**			
ASRS HS	0.53^***^	–		
ASRS total	0.63^***^	0.86^***^	–	
ASRS_score	0.49^***^	0.86^***^	0.74^***^	–

*** *p < 0.001.*

The validity of the ASRS was assessed using the WURS. In Table 2, we show the correlation matrix between the three ASRS values and the WURS. All correlations were large (≥ 0.49). These correlations remained similar when computed separately for each sex, education category, or age category.

## Discussion

We previously showed that ADHD can be considered a lifelong disorder even among the Czech population (Vňuková et al., 2021) and thus confirmed the findings of previous studies such as Montejano et al. (2011) and Ramos-Quiroga et al. (2013).

Currently, in the Czech Republic, the only reliable source of diagnosis is a trained clinician. Although there are recommendations to use, for example, the Czech translation of the Diagnostic Interview for AHD in Adults (DIVA), this is unfortunately not yet common practice among clinicians; we would recommend the increased utilization of the DIVA. Further recommendations advise the use of a screening tool, such as the ASRS or WURS (Cahová et al., 2010). One of the screening tools that is most frequently used worldwide is the ASRS. This tool has been previously translated into the Czech language, but to date, no study has assessed its psychometric properties. This study therefore aimed to assess its psychometric properties on a representative sample of the Czech population. Although ADHD has been exhaustively researched in the Czech Republic by, for example, Paclt (2002, 2007) or Ptacek (2014a), a large-scale study to provide conclusive evidence about the translated version of the ASRS has been missing.

Our current study now shows that similar to other countries (Wyrwich et al., 2016; Kiatrungrit et al., 2017; Takeda et al., 2017; Somma et al., 2019), the Czech Republic now has valid translation of the ASRS tool.

Our analysis provided information on the very satisfactory characteristics of the WURS (Cronbach’s *α* = 0.94, ω_T_ = 0.94) and the satisfactory characteristics of the ASRS (Cronbach’s *α* = 0.89, ω_T_ = 0.88). The psychometric properties of the ASRS are comparable to those reported by Adler et al. (2006): Cronbach’s *α* = 0.88–0.89. The psychometric properties were then checked by correlating both questionnaires, with a satisfactory result (*r* = 0.53, *p* < 0.001). Nonetheless, both of these questionnaires, if used alone, skew toward the overestimation of the presence of ADHD, and we strongly recommend only using them as supplementary tools.

Overall, we showed the good psychometric properties of the ASRS, and we showed that when used as a screening tool in the general population, the full 18-question version slightly outperformed the 6-item screening questionnaire. This finding goes against the recommendation of other researchers who found that the shorter version outperformed the 18-item scale (Kessler et al., 2007). However, in combination with psychiatric assessment together with the recommendation from the authors of ASRS, we can also recommend using the shorter version of the ASRS, which still has overall good psychometric properties.

Overall, our findings indicate not only the good psychometric quality of the ASRS scale but also the possibility of its reliable use in the Czech language.

### Limitations

One limitation of this study was that we did not directly assess ADHD in both childhood and adulthood, and the retrospective assessment of the presence of ADHD in childhood in adults has significant methodological limitations. As we already mentioned, the diagnosis of ADHD was not introduced until 1980, and the average age of the people in the study was 41.56 years. Thus, many of the subjects were at least in adolescence when the criteria were published. Furthermore, the ICD 10 is used in the Czech Republic, which uses different terminology and does not have unambiguously identical criteria for ADHD (American Psychiatric Association, 2013; World Health Organization, 2019). Last, a major limitation of our study was that we did not use clinical interviews to ascertain the presence of adult ADHD. This is because this was a cross-sectional questionnaire study aimed at mapping the possible severity of ADHD symptomatology; however, we realize that for future research, this is recommended.

### Conclusion

To conclude, our results of CFA as well as EFA show that the Czech translation of ASRS has good psychometric properties and that it is comparable to its English original in terms of performance. Nonetheless, we need to be cautious if using it as a standalone tool, as this validation study was performed with the general population. Hence, we strongly recommend using the Czech version of the ASRS only in combination with clinical judgment. For this reason, we believe that the shorter version is sufficient at this time (although the longer version outperforms it). It will help clinicians guide their judgment while not taking too much time to administer and analyze.

For future research, we will focus our efforts on including the analysis of the performance of the ASRS when compared to the clinical assessment. Only after we see how the ASRS performs in these conditions can we recommend its use as a standalone tool.

For now, as a short screening tool, we show that the ASRS has good psychometric properties among the general population and thus prove the validity of the Czech translation. Nonetheless, we are aware that for high-quality diagnosis, we should only use tools that show good psychometric properties in terms of reliability and factor structure. Thus, the shortened version is a promising alternative. It is crucial to emphasize that without proper psychiatric assessment, one cannot make judgments about the diagnosis. This validation of a shortened scale *via* psychiatric assessment is one of the future goals.

## Data Availability Statement

The data sets presented in this article are not readily available because the data set could only be pseudoanonymized. Requests to access the data sets should be directed to Martina Vňuková, martina.vnukova@lf1.cuni.cz.

## Ethics Statement

The studies involving human participants were reviewed and approved by Ethics committee of the General University Hospital, Prague. The patients/participants provided their written informed consent to participate in this study.

## Author Contributions

MV performed the literature search and final write-up of the article. FD performed the statistical analysis and results interpretation. RP and JR performed the study design and development of the questionnaire battery. MG performed the final review and corrections. All authors contributed to the article and approved the submitted version.

## Funding

This work was funded by the GAČR–1811247S, Progres Q 06 1LF and Cooperatio 207031 Health Sciences.

## Conflict of Interest

The authors declare that the research was conducted in the absence of any commercial or financial relationships that could be construed as a potential conflict of interest.

## Publisher’s Note

All claims expressed in this article are solely those of the authors and do not necessarily represent those of their affiliated organizations, or those of the publisher, the editors and the reviewers. Any product that may be evaluated in this article, or claim that may be made by its manufacturer, is not guaranteed or endorsed by the publisher.
